# Foreign Medical Literature

**Published:** 1858-02

**Authors:** 


### FOREIGN MEDICAL LITERATURE.

We know of no quarterly more valuable than *The Medico-Chirurgical Review.* $3 a year. Republished by the Messrs. Wood, of this city.

*Braithewaite's Retrospect*, semi-annually, at $2 a year, by Stringer & Townsend, New York, and *Ranking's Abstract*, by Lindsay & Blakiston, Philadelphia, same price, are invaluable to general practitioners.

The *London Lancet*, $6 a year, monthly, republished by Stringer & Townsend, it is not necessary to notice; it is quoted as authority all over the world.

*Littell's Living Age*, weekly, $6 a year, Boston, was the favorite of John Quincy Adams. No. 714 is, we think, the best yet issued.

*Christian Review*, Baltimore, quarterly, $3 a year, is the ablest periodical in the Baptist Church that has ever come under our notice. *Sargent's School Monthly*, Boston; *American Agriculturist*, New York; *Scientific American; The Happy Home; Country Gentleman;* all are sterling publications.

